# Unveiling the Potential of Extracellular Vesicles as Biomarkers and Therapeutic Nanotools for Gastrointestinal Diseases

**DOI:** 10.3390/pharmaceutics16040567

**Published:** 2024-04-21

**Authors:** Valentina Arrè, Rita Mastrogiacomo, Francesco Balestra, Grazia Serino, Federica Viti, Federica Rizzi, Maria Lucia Curri, Gianluigi Giannelli, Nicoletta Depalo, Maria Principia Scavo

**Affiliations:** 1 National Institute of Gastroenterology, IRCCS de Bellis, Via Turi 27, 70013 Castellana Grotte, Italy; valentina.arre@irccsdebellis.it (V.A.); francesco.balestra@irccsdebellis.it (F.B.); grazia.serino@irccsdebellis.it (G.S.); gianluigi.giannelli@irccsdebellis.it (G.G.); 2 Department of Chemistry, University of Bari, Via Orabona 4, 70125 Bari, Italy; rita.mastrogiacomo@uniba.it (R.M.); marialucia.curri@uniba.it (M.L.C.); 3 Institute for Chemical-Physical Processes (IPCF)-CNR SS, Via Orabona 4, 70125 Bari, Italy; f.rizzi@ba.ipcf.cnr.it; 4 National Interuniversity Consortium of Materials Science and Technology (INSTM), Bari Research Unit, 70126 Bari, Italy; 5 Institute of Biophysics—National Research Council (IBF-CNR), Via De Marini 6, 16149 Genova, Italy; federica.viti@ibf.cnr.it

**Keywords:** extracellular vesicles, biomarkers, delivery of therapeutic compounds, gastrointestinal diseases

## Abstract

Extracellular vesicles (EVs), acting as inherent nanocarriers adept at transporting a range of different biological molecules such as proteins, lipids, and genetic material, exhibit diverse functions within the gastroenteric tract. In states of normal health, they participate in the upkeep of systemic and organ homeostasis. Conversely, in pathological conditions, they significantly contribute to the pathogenesis of gastrointestinal diseases (GIDs). Isolating EVs from patients’ biofluids facilitates the discovery of new biomarkers that have the potential to offer a rapid, cost-effective, and non-invasive method for diagnosing and prognosing specific GIDs. Furthermore, EVs demonstrate considerable therapeutic potential as naturally targeted physiological carriers for the intercellular delivery of therapeutic cargo molecules or as nanoscale tools engineered specifically to regulate physio-pathological conditions or disease progression. Their attributes including safety, high permeability, stability, biocompatibility, low immunogenicity, and homing/tropism capabilities contribute to their promising clinical therapeutic applications. This review will delve into various examples of EVs serving as biomarkers or nanocarriers for therapeutic cargo in the context of GIDs, highlighting their clinical potential for both functional and structural gastrointestinal conditions. The versatile and advantageous properties of EVs position them as promising candidates for innovative therapeutic strategies in advancing personalized medicine approaches tailored to the gastroenteric tract, addressing both functional and structural GIDs.

## 1. Introduction

Gastrointestinal diseases (GIDs) encompass a broad spectrum of both benign and malignant pathologies affecting the digestive tract, including the accessory organs of digestion such as the liver, biliary tract, and pancreas [1]. The GIDs can be classified into two main groups, functional and structural, characterized by common symptoms such as diarrhea, gastrointestinal bleeding, abdominal pain, nausea, heartburn, vomiting, constipation, and bloating [1].

*Functional GIDs*: The functional GIDs are represented by irritable bowel syndrome (IBS), associated with nausea, bloating, constipation, diarrhea, or uncomplicated gastroesophageal reflux disease (GERD).

*Structural GIDs:* The structural GIDs consist in various conditions, such as viral and autoimmune hepatitis, acute and chronic pancreatitis, peptic ulcer disease in gastric and duodenal regions, cholelithiasis, complicated GERD, such as diverticular disease, and inflammatory bowel diseases (IBD), including Crohn’s disease (CD) and ulcerative colitis (UC). Additionally, both benign and malignant neoplasms are considered within the category of structural GIDs [2].

In severe cases, GIDs can lead to debilitating symptoms, significantly impacting the quality of life for affected individuals. Depending on the diagnosis, current treatment options include lifestyle modifications, dietary interventions, gut microbiota manipulation, and the use of specific drugs [3]. However, the therapeutic response is often suboptimal, leading to the need for GID treatment optimization. As a result, there is a growing interest in investigating new therapeutic approaches.

In this context, extracellular vesicles (EVs) are emerging as a promising area of research. EVs are lipid bilayer-enclosed vesicles secreted by most cells, originating from a diverse array of sources. In mammals, EVs have been identified across a wide spectrum of biological samples, including tissues, bodily fluids (such as blood, urine, saliva, milk, amniotic fluid, cerebrospinal fluid, etc.), and cell cultures derived from both human and non-human origins. Moreover, EVs have been effectively also isolated from plants (e.g., fruits, leaves, and roots), as well as from other organisms, including bacteria, fungi, and parasites [4,5,6]. EVs originating from diverse cellular sources exhibit unique characteristics and functions, necessitating a comprehensive classification scheme. This following classification delineates EVs derived from eukaryotic cells (including humans, other mammals, and plants) and prokaryotic cells (including archaeal bacteria).

### 1.1. Prokaryotic Cell-Derived EVs

*Bacterial EVs (BEVs):* Both Gram-negative and Gram-positive bacteria secrete EVs, commonly referred to as bacteria-derived EVs (BEVs). BEVs produced by Gram-negative bacteria can be categorized into three types: outer membrane vesicles (OMVs), outer-inner membrane vesicles (O-IMVs), and explosive outer membrane vesicles (E-OMVs). In contrast, Gram-positive bacteria possess a single-cell membrane encased by a robust cell wall, and their EVs consist of cytoplasmic membrane vesicles. BEVs play diverse roles including virulence, biofilm formation, and antibiotic resistance, carrying a cargo comprising proteins, lipids, nucleic acids, and virulence factors. They are integral components of the microbiota, influencing microbial communication, community dynamics, and host-microbe interactions [7].

*Archaeal EVs:* Archaeal EVs (AEVs) exhibit differences in biogenesis and the taxa of origin, with crenarchaeotal AEVs (C-AEVs) formed via the archaeal ESCRT machinery and euryarchaeotal AEVs (E-AEVs) produced through cell membrane blebbing. Similar to bacterial OMVs, these EVs function in intercellular communication, stress response, and survival strategies within archaeal cells [8].

### 1.2. Eukaryotic Cell-Derived EVs

#### Mammalian Cells

The EVs derived from mammalian cells are canonically classified on the basis of their dimension, and recently, also by the origin and characteristic proteins used as specific EV markers (Table 1).

*i. Exosomes:* Exosomes are small EVs (30–150 nm) originating from the endosomal system. They encapsulate diverse cargo, including proteins (notably the tetraspanin family and heat shock proteins), lipids, and nucleic acids, facilitating intercellular communication and modulating various physiological processes.

*ii. Microvesicles or Ectosomes (including small ectosomes):* These vesicles bud directly from the plasma membrane and exhibit a broad size range (50–1000 nm). *Small ectosomes*, a subset of microvesicles, typically range from approximately 50 to 200 nm in diameter. They share many similarities with larger microvesicles but are characterized by their smaller size. Microvesicles carry a cargo reflective of the parental cell membrane and participate in cell signaling, immune response modulation, and tissue repair.

*iii. Apoptotic Bodies:* Generated during apoptosis, apoptotic bodies are larger vesicles (>1000 nm) containing fragmented DNA and cellular organelles. They contribute to the clearance of apoptotic material and immune regulation.

**Figure 1:**
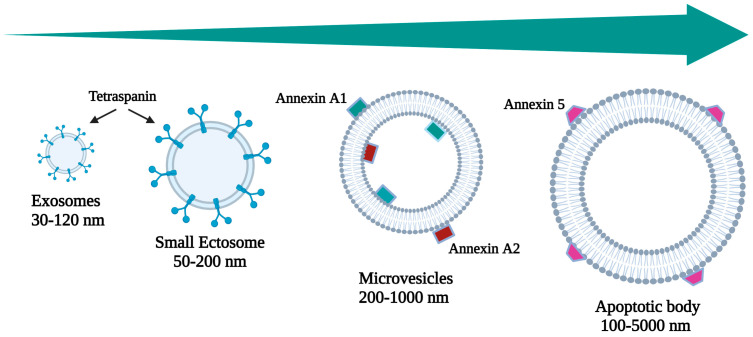
Schematic size-based classification of EVs secreted by mammalian cells.

Figure 1 illustrates a schematic size-based classification of EVs secreted by mammalian cells, while Table 1 provides the classification of EVs released from mammalian cells, categorized according to their definition, dimensions, origin, and molecular expression markers.

### 1.3. Plant-Derived EVs

The lack of definitive understanding regarding the biogenetic pathways of plant-derived extracellular vesicles typically precludes their classification using mammalian vesicle classification methods. Additionally, a lack of uniform and standardized nomenclature exists in the relevant literature. Throughout this review, the term “plant-derived extracellular vesicles” (PDEVs) will be consistently employed. Released by plants, PDEVs constitute heterogeneous populations of vesicles harboring diverse functions, primarily originating from multivesicular bodies (MVBs), autophagosomes, vacuoles, and exocyst-positive organelles (EXPOs). The size range of these nanovesicles typically falls between 50 and 1000 nm, with variations observed depending on the specific plant source [16]. PDEV biogenesis occurs in several phases of blebbing, budding, and shedding of plasma membranes or the exocytosis of multivesicular bodies [17].

### 1.4. EVs for Therapeutic or Diagnostic Purposes

Each category of EVs exhibits unique characteristics, cargo composition, and functional roles. EVs serve as carriers for various active biomolecules, both as internal cargo and on their surfaces, facilitating their transfer from donor cells to recipient cells. Consequently, they play crucial roles in numerous intercellular signaling pathways, modulating diverse physiological and pathological cellular processes [6].

Due to their capacity to transport various bioactive molecules, such as proteins, lipids, genetic material, and metabolites, EVs present a significant potential role in the diagnosis, monitoring, and prognostic estimation of diseases. They can serve as tools for detecting clinically relevant molecular markers from biofluids through liquid biopsy, which entails the non-invasive analysis of biofluids (e.g., blood, urine) to acquire disease-related information [18,19,20].

Given their ability to transport bioactive molecules and traverse biological barriers within the body, including the mucous barrier in the gastrointestinal tract, EVs present promising therapeutic avenues. These innovative perspectives extend to the treatment of GIDs, capitalizing on EVs capability to cross physiological barriers and deliver therapeutic payloads to the target tissues. In contrast to synthetic nanoparticles, which often provoke adverse immunological reactions such as cytokine release syndrome, cross-reactivity with endogenous proteins, anaphylaxis, the neutralization of biological activity, and non-acute immune responses, EVs derived from both human and non-human cells are distinguished by their high biocompatibility, stability, very low immunogenicity, and overall safety profile [21,22,23]. These findings support the idea of utilizing EVs as drug carriers, emphasizing their potential safety and biocompatibility.

For therapeutic applications, it is worth noting that both *i.* naturally occurring EVs carrying endogenous cargos known for their beneficial properties and *ii.* artificially engineered EVs loaded with therapeutic exogenous cargo can be explored.

For instance, intact PDEVs retaining their natural structural integrity and endogenous bioactive cargo, following simple isolation from plants, hold the potential to alleviate the pathological conditions in various species, including humans. They offer diverse therapeutic alternatives such as anti-infection, anti-inflammation, anti-tumor, anti-aging, and more [24]. Furthermore, Zheng et al. demonstrated the therapeutic potential of BEVs derived from *Akkermansia muciniphila* in reducing mucosal damage, increasing MUC2 expression, and subsequently improving mucus integrity, leading to a reduction in the intestinal permeability in mice with colitis [25]. Similarly, BEVs derived from *Bifidobacterium acidifaciens* were shown to induce the repair of mucosal damage and achieve complete restoration of the mucus and gut microbiota balance in mice with dextran sulfate sodium (DSS)-induced colitis [26].

In an alternative approach, numerous studies have showcased the feasibility of skillfully engineering EVs for their utilization as natural delivery nanosystems for exogenous therapeutic agents. By genetically modifying EV-producing cells or manipulating purified EVs, it becomes feasible to encapsulate therapeutic molecules within EVs. For instance, in a study involving the intravenous administration of EVs purified from bovine milk to mice and loaded with chemotherapeutic/chemo preventive agents, no adverse events were induced, and only moderate cytokine release was observed [27].

Furthermore, the surface membrane of EVs can be engineered for tissue-specific peptides or ligands, enabling them to be directed to specific cells by displaying targeting moieties on their surface [28]. Interestingly, this manipulation enables enhanced organ or cell-specific targeting, thereby bolstering the therapeutic effectiveness of EVs in treating various specific pathologies [29,30,31].

EVs originating from different tissues have been shown to exhibit specific homing capabilities. Central nervous system-derived EVs possess the ability to traverse the blood-brain barrier (BBB) and function as a distinctive drug delivery system targeting specific neuron populations. Additionally, EVs derived from microglial cells exhibit the capability to target conditions such as multiple sclerosis and chronic inflammatory diseases of the central nervous system [32]. Notably, EVs derived from cancer cells demonstrate a selective tropism for the tumor tissue from which the vesicles originated [33]. This offers an opportunity to leverage a naturally occurring biological process for therapeutic applications. Indeed, while unmodified EVs may contribute to the formation of metastases in healthy tissues, it is evident that cancer cell-derived EVs engineered for the delivery of exogenous therapeutic compound possess the capability to target their parental tumor cells and exert therapeutic activity when properly modified. This suggests a potential avenue for delivering anti-cancer drugs to specific organs [34].

Interestingly, the utilization of EVs in an autologous manner involves transferring patient cells into culture medium, isolating vesicles, loading them with therapeutic agents, and subsequently re-administering them to the patient. This approach has been proposed as a strategy for the development of new personalized treatments for diseases [35].

Hence, the targeted therapeutic potential of EVs can be realized either through the innate targetability of EVs, achieved by selecting specific EV donors, or through the acquired targetability of EVs, achieved via bioengineering of the EV surface.

**Figure 2:**
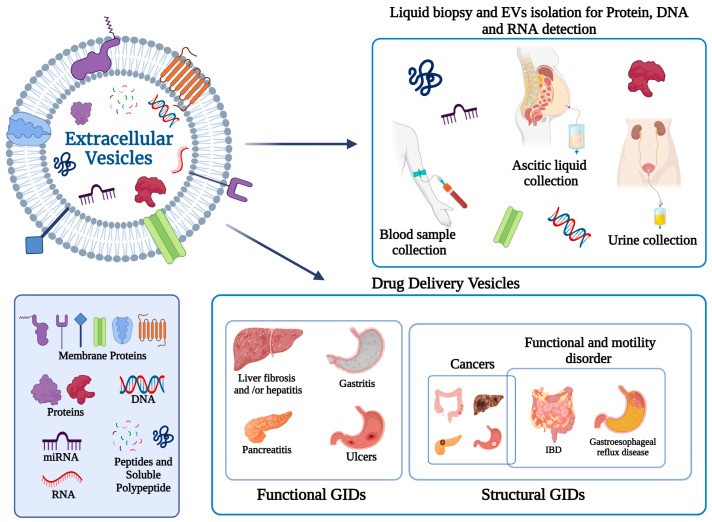
Schematic representation of EVs and their use for diagnostic and therapeutic purposes in GIDs.

The aim of this review is to *i.* underscore the potential of EVs delivering their endogenous cargo as biomarkers in GIDs, and *ii.* explore the fundamentals of the emerging concept of EVs as drug delivery nanotools, with a specific emphasis on their application in gastroenteric disease (Figure 2).

The utilization of EV-derived biomarkers introduces a novel dimension to the field, providing non-invasive and potentially more accessible diagnostic alternatives compared to conventional techniques (such as endoscopy, CT, and ultrasound), which are considered the gold standard in the clinical diagnosis for GIDs.

The EV-based therapeutic approach shows significant promise in advancing personalized medicine strategies tailored to the gastroenteric tract, addressing both functional and structural GIDs.

## 2. EVs as Mediators in Gastroenteric Pathogenesis and Biomarkers of GIDs

EVs play an endogen crucial role in both the pathogenesis of diseases, as well as in the maintenance of system and organ homeostasis. Indeed, physiological, and pathological EVs act as intracellular messengers, transporting cargo, including pathologically active biomolecules, to both local and distant tissues. The multifaceted roles of EVs in the gastrointestinal tract involve the active participation of various cell types, including intestinal epithelial cells, endothelial cells, and immune cells. Microbiota-derived EVs have to be also considered as important negative or positive players in regulating the intestinal microenvironment [36]. Indeed, various gut-related issues encompassing intestinal infections, IBD, metabolic dysregulation, and even cancers, can arise due to an imbalance in the production of microbiota-derived EVs [37]. In health conditions, EVs play a crucial role in regulating the homeostasis of anti-inflammatory conditions, influencing gut microbiota composition, the functionality of vascular and epithelial barriers, and stimulating immune cells [38]. Their active beneficial involvement contributes significantly to the preservation of intestinal homeostasis, mitigation of intestinal inflammation, and modulation of systemic metabolism. In pathological conditions, EVs play a significant role in the spread of diseases, including within the gastroenteric apparatus. They not only have the capability to alter gene expression in target cells through their mRNA or miR cargo [39] but also crucially contribute to modulating the microenvironment of GIDs. For example, they are involved in modifying the tumor microenvironment, triggering processes such as angiogenesis [40] and metastasis, and establishing hypoxia [31,41].

### 2.1. Aim of the Review

In this section, various and recent studies that emphasize the substantial involvement of EVs in the progression of different pathologies in the gastroenteric tract will be explored. These studies shed light on the mechanisms that actively involve EVs and are related to carcinogenesis, inflammation processes, or chronic abdominal pains, underscoring the significance of EVs as excellent biomarkers for several GIDs. Indeed, given that EVs act as nanocarriers of pathological bioactive molecules, their isolation from patients’ biofluids enables the characterization of their cargo, thus identifying biomarkers that potentially may provide fast, low-cost, and non-invasive approaches to diagnose and prognose specific GIDs.

### 2.2. EVs in Liver Pathogenesis

In liver-related GIDs, specific proteins from the frizzled (FZD) family, namely FZD1 and FZD7, associated with the Wnt pathway, have been implicated in contributing to the heightened hepatic fibrosis and inflammation in metabolic dysfunction-associated steatotic liver disease (MASLD). These proteins have been identified in the exosomes isolated from the plasma of MASLD-affected subjects [42,43]. In the context of MASLD, recent findings have highlighted an imbalance of specific lipids, specifically oleic and palmitic acid, delivered within circulating exosomes. The relative levels of palmitic acid to oleic acid within these exosomes play a crucial role in MASLD degeneration. When the exosomes carry a higher concentration of palmitic acid compared to oleic acid, the hepatic cells that endocytose these vesicles activate the necroptosis pathway, leading to cell death. Conversely, restoring the balance of these two acids in exosomes from MASLD patients prevents cell death [44]. Therefore, the use of a lipidomic analysis of plasma-derived exosomes could be a good diagnostic and prognostic approach in patients with MASLD.

Recently, changes in lipid membranes have been observed through the exosomal fraction of EVs derived from patients affected by MASLD before the occurrence of hepatic fibrosis detectable by performing a fibroscan. These modifications in exosomal membranes influence the membranes of stellate cells (LX2) stimulated by the exosomes from these patients. This suggests a potential role in the early detection of disease progression, which could be discerned through the lipidomic analysis of exosomal membranes [44,45]. In the realm of liver pathologies, the analysis of circulating EVs has proven effective in distinguishing patients with metabolic dysfunction-associated steatohepatitis (MASH) from those with chronic hepatitis compared to healthy controls. Notably, in various studies, two miRs found in serum EVs, namely miR-122 (a major hepatic miR) and miR-192, have been demonstrated to increase with the progression of activity and the development of fibrosis [46]. Thietart et al. conducted research on the utility of EVs as biomarkers for alcoholic hepatitis. They found that Cytokeratin 18 contained within the EVs served as a diagnostic marker with higher specificity (81%) and sensitivity (76%) [47]. As the pathology progressed to cirrhosis, other studies indicated, in large EVs, an increase of platelet-derived growth factor B (PDGF-b), which could be associated with an instrumental fibrosis diagnosis [48]. The prediction of a poor prognosis is crucial for ensuring the correct therapy with higher impact efficacy. A significant study focused on hepatocyte-derived EVs demonstrated their ability to predict 6-month mortality by evaluating the variation of CD144+ and CD62E+ molecules carried by EVs. This predictive capability was observed independently of the Child-Pugh (the score for cirrhosis mortality estimates cirrhosis severity) and MELD scores (Model for End-Stage Liver Disease is validated as a predictor of survival in patients with cirrhosis, alcoholic hepatitis, acute liver failure, and in patients with acute hepatitis) [49].

EVs play a relevant role in the evolution of hepatocellular carcinoma (HCC) [50]; indeed, in EVs derived from the serum of patients with HCC, the presence of miR-21 is elevated compared to healthy subjects, thereby identifying miR-21 as a diagnostic biomarker for HCC [51,52]. Another miR-12-92 cluster originating from the EVs of tumor-associated macrophages significantly has been found to contribute to the dysregulation of the TGF-β1/BMP-7 pathways in HCC cells. This leads to the promotion of invasion and metastasis in HCC through the inhibition of the TGFBR2/Smad ubiquitylation regulatory factor 1 (Smurf1)/activin A receptor type 1 (ACVR1) signaling pathway [53].

### 2.3. EVs in Pancreas Pathogenesis

In pancreas-related GIDs, various studies have highlighted the involvement of EVs in conditions such as autoimmune pancreatitis, chronic pancreatitis, and acute pancreatitis. These studies suggest that EVs play a role in the development and progression of pancreatic diseases by utilizing their biological capability to fuse with the plasma membrane of recipient cells and deliver their contents, which include transcription factors, oncogenes, microRNAs (miRNAs), and messenger RNAs (mRNAs), into recipient cells [54].

During acute pancreatitis, studies have demonstrated that circulating exosomes released by the pancreas into the pancreatitis-associated ascitic fluid (PAAF) can migrate to alveolar compartments and activate macrophages. Particularly noteworthy is the identification of two distinct populations of exosomes during acute pancreatitis, characterized by significant differences in cell distribution, protein content, and miRNA composition. These differences lead to varied physiological effects and implications associated with each exosome population. During pancreatitis, plasma-derived exosomes exhibit high levels of the inflammatory miRNA miR-155, while showing low levels of miR-21 and miR-122. In contrast, proteomic analysis revealed that PAAF exosomes contain higher concentrations of histones and ribosomal proteins compared to plasma exosomes [55]. Recently, a prospective study aimed at reviewing the diagnostic performance of EV biomarkers for pancreatic cancer (PC) has demonstrated that combining EV RNAs with EV proteins enables highly efficient diagnosis [56].

The early detection of pancreatic ductal adenocarcinoma (PDAC) poses a challenge due to the late onset of symptoms and limited visibility of sub-centimeter cancers upon imaging. EVs derived from pancreatic juice (PJ) and loaded with miRNAs have emerged as excellent biomarkers. Nesteruk et al. highlighted the utility of certain EV-associated miRNAs, such as EV-miR-21, EV-miR-25, and EV-miR-16, which show increased levels in PDAC cases compared to controls in PJ. Additionally, EV-miR-210 was found to be increased only in serum. The combined use of both PJ EV-associated miRNAs and serum EV-associated miRNAs, along with the traditional biomarker, CA 19-9, yielded a specificity of 84.2% and a sensitivity of 81.5% [57]. A notable study conducted by Flammang et al. demonstrated the prediction of PDAC with a diagnostic accuracy comparable to CA 19-9 using the expression of EV miR-192-5p. In a recent study by Yang et al., a wide panel of 22 EV biomarkers, including MUC1, MUC2, MUC4, MUC5AC, MUC6, Das-1, STMN1, TSP1, TSP2, EGFR, EpCAM, GPC1, WNT-2, EphA2, S100A4, PSCA, MUC13, ZEB1, PLEC1, HOOK1, PTPN6, and FBN1, was employed to detect the development of pancreatic cancer in a plasma-based discovery [58,59,60,61,62].

### 2.4. EVs in Gastric Pathogenesis

In gastric pathologies, similar to liver and pancreatic pathologies, EVs have been implicated in the modulation of both oncological and chronic conditions. This involvement has facilitated the identification of molecules contained in EVs that can be useful for monitoring diseases. In GERD, the expression of serum exosomal miR-29a-3p was found to significantly increase in rats with chronic GERD, but not in rats with gastric ulcer or colitis. This suggests that serum exosomal miR-29a-3p may serve as a specific marker for chronic reflux esophagitis (RE) [63].

*Helicobacter pylori* (HP) gastritis is an induced disease that can progress to chronicity if HP is not eradicated. The chronicization process is attributed not only to the bacterium’s ability to replicate and survive in the gastric environment, but also, notably, to the capability of gastric mucosa cells to transfer bacterial toxins into recipient cells via endogen EVs. This transfer leads to the induction of IL-8 production and subsequent morphological cellular changes [64], potentially contributing to the induction of malignancy. As a result, it has been reported that the resident cells and their EVs serve as mediators in relation to the induced pathology [65].

Chronic atrophic gastritis (CAG) is considered a precancerous stage of intestinal-type gastric cancer (GC), and its diagnosis and follow-up traditionally rely on endoscopic and histopathological evaluation [66]. However, in recent years, a new non-invasive diagnostic method has emerged, complementing existing approaches. This method involves the use of small RNA sequencing (sRNA-Seq) to analyse miRNA profiles in the serum exosomes of CAG patients. Liu et al. identified a significant upregulation of exosomal miRNA, namely hsa-miR-122-5p, in CAG patients. This finding suggests that hsa-miR-122-5p holds promise as a biomarker for CAG [67].

Recently, several EV proteins, miRNAs, and long non-coding RNAs (Lnc-RNAs) have shown great potential for the non-invasive diagnosis and prognosis of GC [68]. Among these biomarkers, one protein identified as a potential marker and found in exosomes derived from gastric juice is BarH-Like2 homeobox protein (BARHL2). BARHL2 exhibits a high level of methylation, demonstrating a sensitivity of 90% and specificity of 100% in distinguishing between GC patients and healthy subjects [69]. Additionally, two other promising markers for the early diagnosis of GC are LncUEGC1 and LncRNA HOTTIP, which were found to be transported by plasma exosomes in GC patients. An upregulation of LncRNA HOTTIP was observed in 126 GC patients compared to 120 healthy subjects [70,71]. Furthermore, similar to observations in the liver, an increase in FZD10, expressed on the exosomal membrane, has been demonstrated to be implicated in the growth and spread of GC. The presence of FZD10 on exosomes correlates with its presence in cancer tissue and is directly associated, via phospho-ERK1/2, with tissue Ki-67 levels [18].

### 2.5. EVs in Intestinal Pathogenesis

In chronic diseases, such as those affecting the gastroenteric tract, like IBD, EVs play a crucial role. A non-invasive analysis could be facilitated by the use of salivary exosomes. Recent findings indicate that salivary exosomes containing proteasome alpha subunit type 7 (PSMA7) showed a remarkable increase in patients with active IBD, making it a promising biomarker to be integrated into diagnostic tools in clinical settings [72]. Additionally, nuclear paraspeckle assembly transcript 1 (NEAT1), a protein involved in macrophage polarization, and NEAT1 lncRNA transported by EVs have been found at high levels in the sera of active IBD patients. NEAT1 lncRNA is implicated in modulating the inflammatory response and regulating intestinal barrier function [73]. Another lncRNA, named lncRNA H19, has been identified as a potential diagnostic biomarker for IBD due to its overexpression and conveyance in EVs [74]. Similarly, high levels of annexin 1 were found in the EVs derived from the sera of IBD patients, suggesting its potential as a biomarker for intestinal mucosal inflammation in IBD [75].

Furthermore, miRs encapsulated in EVs and involved in modulating IBD, could serve as novel biomarkers to monitor disease progression [76]. In acute colitis models, EVs-miR-200b-3p, a member of the miR-200 family known to regulate epithelial-mesenchymal transition (EMT), was significantly increased, suggesting its potential as a biomarker for active disease [77].

As observed in GC, the FZD10 protein was also detected in plasma-derived exosomes from patients affected by colorectal cancer (CRC), indicating its potential as a CRC biomarker [18,20,78]. In addition, the Wnt protein, a ligand of FZD10, has been demonstrated to be transported by exosomes and to be implicated in drug resistance in differentiated CRC cells, potentially correlating with shortened patient survival [79].

Another protein evaluated using ELISA directly on the plasma exosomes of CRC patients is Copine III (CPNE3). Combined with a carcinoembryonic antigen (CEA), this approach achieved 84.8% sensitivity and 81.2% specificity, thereby procuring an optimal diagnostic and prognostic tool [80]. Novel biomarkers for the non-invasive diagnosis and prognosis of CRC have been found in feces-derived EVs, including CD147 and A33, which offer a clinical sensitivity of 89% compared to the commonly used serum biomarker for CRC diagnosis, a carcinoembryonic antigen (CEA) [81]. Furthermore, in EVs from metastatic CRC patients, myristoylated alanine-rich protein kinase C substrate-like protein 1 (MARCKSL1) has been identified as a potential biomarker, showing higher levels in patients with metastatic CRC compared to non-metastatic CRC and healthy individuals [82].

A summary of the biomarkers involved in various gastroenteric pathologies is provided in Table 2.

## 3. Functional Gastrointestinal Diseases and EV-Based Therapy

Functional gastrointestinal disorders, such as irritable bowel syndrome and functional dyspepsia, represent prevalent conditions associated with a markedly diminished quality of life and extensive healthcare utilization. Excluding germline genetic causes, these non-neoplastic disorders arise from dysregulations in gastrointestinal functioning, encompassing alterations in gut sensitivity, motility, microbiota composition, immune responses, central nervous system processing, chronic stress, and some medications. Persistent symptoms throughout the gastrointestinal tract, including pain, dyspepsia, and irregular bowel habits, characterize these disorders. Maladaptive patient behaviors, stress, and psychological comorbidity exacerbate these chronic symptoms [85]. GERD commonly exhibits symptoms such as heartburn and regurgitation; however, it can also manifest in atypical or extraesophageal symptoms. These atypical symptoms may include asthma, chronic cough, laryngitis, hoarseness, chronic sore throat, dental erosions, and noncardiac chest pain.

As EVs play a crucial role in mediating inflammation, immune responses, the maintenance of gut barrier integrity, and overall intestinal homeostasis, there has been a growing exploration of the application of EVs in the treatment of functional GIDs (Table 3).

### 3.1. EVs in Liver Disease Therapy

EVs derived from erythrocytes possess a natural propensity to accumulate in the liver, contributing to a favorable safety profile [86]. Specifically, the presence of integrin α_v_β_5_ is associated with the liver tropism of EVs [87], while the presence of EpCAM defines their localization in intestinal epithelial cells, maintaining immune balance [88]. In the context of inflammatory GIDs, EVs derived from intestinal microbiota hold promise for reducing adipose dysfunction and reversing obesity and inflammation [89].

EVs have emerged as effective targeted drug delivery systems for treating hepatitis, a condition characterized by liver inflammation and damage. Notably, EVs released by mesenchymal stem cells (MSCs) have been used for liver disease treatment, leading to regeneration in several cases [90]. For instance, EVs derived from human umbilical cord mesenchymal stem cells (Huc-MSCs) have been employed to treat liver fibrosis by reducing transforming growth factor-beta 1 (TGF-β1) expression and inhibiting liver EMT in vivo. The effective dosage of EVs required to restore liver status was determined to be 250 μg, administered through direct injection into the liver lobes [91]. The protective mechanism of Huc-MSC in liver failure involves the enrichment of miR-455-3p in EVs, which plays a crucial role in inhibiting the activation of cytokines produced by infiltrated macrophages in local liver damage [92]. Furthermore, MSC-derived EVs, in combination with the drug, Nilotinib, have shown efficacy in reducing steatosis in the treatment of liver fibrosis [93].

Studies have also demonstrated the inhibition of the NLRP3 pathway during liver inflammation by EVs derived from Huc-MSC, pre-treated with TNF-α, thereby promoting liver repair processes [94] as drug delivery systems for treating other hepatic diseases, such as fulminant hepatitis and virus infections. A preventive approach against hepatitis C has been developed, involving the testing of an EV-based vaccine on mice. In particular, the DNA plasmids were used to generate modified retrovirus-like EVs, aiming to confer immune protection against HCV [95].

MASLD stands out as a multifactorial and chronic liver ailment, affecting over 30% of the global population, and lacking a well-defined therapy as of yet [96]. Lifestyle modifications and weight loss are recognized for their potential to ameliorate the syndrome [97]. Recent findings indicate a connection between the progression of MASLD and an imbalanced diet, specifically the disproportionate intake of palmitic acid delivered by exosomes over oleic acid. This dietary imbalance hastens MASLD development into cirrhosis [98]. Scavo et al. demonstrated that rebalancing the fatty acid composition in liver cells, using exosomes derived from MASLD patients loaded with oleic acid, can reverse MASLD degeneration. This approach inhibits the enzymatic cascade involved in disease progression and reduces the fibrotic state [44].

### 3.2. EVs in Gastritis and Esophagitis Therapy

EVs have gained attention as potential therapeutic tools also in gastric functional disorders, such as gastritis, ulcers, and oesophageal reflux. Though the research in this area is still unexplored, therapies based on EVs hold promise owing to their capability to deliver bioactive molecules and modulate cellular processes. Investigations into the therapeutic potential of Bone Marrow Stromal Cell (BMSC)-derived EVs reveal beneficial effects in conditions such as 2,4,6-trinitrobenzene sulfonic acid (TNBS)-induced colitis. This is particularly significant as colitis is often implicated in gastric functional disorders like functional dyspepsia, irritable bowel syndrome (IBS), and esophagitis reflux. The observed therapeutic benefits include the regulation of inflammation, suppression of oxidative stress, and abatement of apoptosis [99]. EVs can convey anti-inflammatory cytokines, miRs, and si-RNA which can modulate the immune response and reduce inflammation. As previously reported, several studies evaluated modified exosomes derived from vaccine milk (mExo) as a novel oral delivery system of si-RNA by combining the intrinsic casein chelation with ultracentrifugation and chromatography methods, to obtain high-yield and purified exosomes. This method has been further developed by fabricating PEG-coated mExo to improve their stability, to avoid their degradation in the stomach, and significantly enhance their permeability through intestinal mucin [100].

In recent years, it has been found that PDEVs can potentially help overcoming the limit of oral drug administration. Indeed, PDEVs present physical features that can enhance drug delivery, especially through oral administration [101]. The utilization of EVs derived from medicinal plants, exemplified by *Kaempferia parviflora* (KP), has shown intriguing potential in the treatment of gastric inflammation. This was demonstrated in a mouse model of ulcers induced by *H. pylori*, where EVs derived from KP contributed to the enhancement of mucosal regeneration [102].

Other diseases of the gastrointestinal tract involve the oesophagus, whose physiology limits the delivery of specific drugs. Hence, a new generation of EV application has been developed as biological pro-regenerative nanotherapeutic agents in esophagitis. Recently, the autologous EVs from adipose-tissue-derived stromal cells, have been administrated in a porcine fistula model by applying thermo-responsive gel directly into the entire fistula. This approach has been found to significantly reduce fibrosis and inflammation, and increase angiogenesis, although it is a minimally invasive strategy in the therapeutic fistula model [103].

The research on the use of EVs for the treatment of GERD is in its early stages, and specific therapies based on EVs are not yet established for clinical use. However, the potential application of EVs in managing conditions like GERD can be explored. EVs anti-inflammatory capabilities represent one of the crucial aspects in the regulation of gastroesophageal reflux, with a potential action on the modulation of the inflammatory responses associated with GERD. Several studies have demonstrated that miRs contained within human umbilical cord mesenchymal stem cell (MSC)-derived exosomes can inhibit the expression of Enamelin. Enamelin is strongly expressed in GERD and in the cells associated with gastroesophageal cancer [104]. In addition, the role of exosomes in GERD has been reported in other recent studies conducted on rats. Additionally, the regenerative properties of EVs, including exosomes, are being explored in various contexts, including tissue repair. In the context of GERD, where chronic exposure to stomach acid can damage the tissues in the oesophagus, the regenerative properties of EVs may be relevant. EVs have the potential to promote tissue repair and protect the oesophageal lining, providing a potential avenue for therapeutic intervention. This area of research holds significant promise for the development of novel approaches to manage and treat GERD and its associated complications.

In the light of such evidence, the EV-based therapies for functional gastric and oesophageal disorders have great potential, although several challenges need to be addressed to finally achieve standard clinical treatments for gastric functional disorders.

### 3.3. EVs in Colitis Therapy

Colitis, also belonging to the functional GIDs, refers to the inflammation of the colon or large intestine, and it can cause a range of symptoms, including abdominal pain, diarrhoea, and rectal bleeding. In this context, recent research has explored the potential of using EVs as drug delivery vehicles. The gut microbiota, which constitutes a diverse community of microorganisms residing in the digestive tract, plays a pivotal role in the development and progression of colitis. Imbalances in the composition of gut bacteria have been consistently linked to the occurrence of these inflammatory conditions [105]. In particular, several studies report the PDEVs use, namely ginger-derived EVs containing miRs, that target several genes in *Lactobacillus rhamnosus* [106]. It has been reported that the miRs in ginger-based EVs target a specific gene or enzyme, called monooxygenase ycnE, suggesting their capability to modulate the expression of these genes, with an increase in indole-3-carboxaldehyde, a ligand of the arylhydrocarbon receptor (AhR) [107]. The AhR is a transcription factor that participates in various cellular processes, notably immune responses. Its involvement includes the upregulation of IL-22, a cytokine that has been associated with the induction of colitis in mouse models [108].

**Figure 3:**
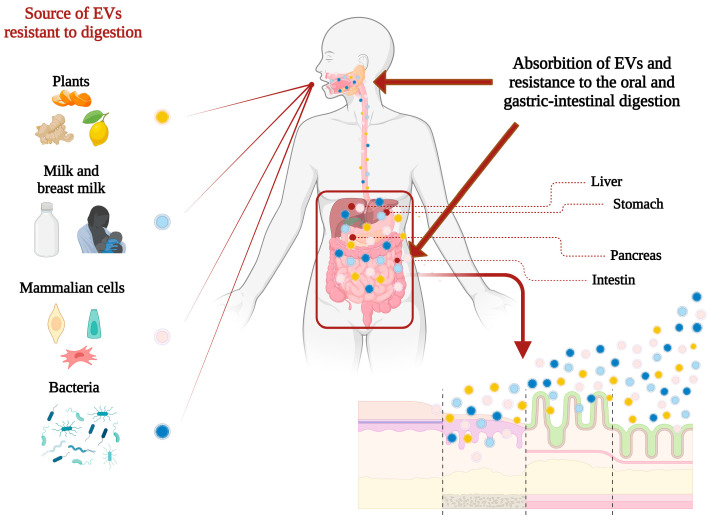
Source of EVs resistant to digestion and absorption in gastrointestinal tract.

EVs have the potential to influence the communication between the gut and the central nervous system, which is central in the pathophysiology of functional gastrointestinal disorders. This crucial interaction is referred to as the gut-brain axis [109]. EVs may transport signalling molecules that contribute to regulating this interaction and managing the associated symptoms. In addition to PDEVs, there are now chemically modified EVs that can be utilized in oral administration. A proposed mechanism for EV absorption in intestinal epithelial cells is transendocytosis [110] or paracellular translocation via the epithelial barrier [111]. A study demonstrated an improvement of pharmacokinetic parameters of orally administered of cow’s-milk-derived EVs modified with paclitaxel (PAC) to increase its stability in circulation in nude mice [112]. The authors proved that orally administered PAC-loaded EVs had lower systemic and immunological toxicities compared to intravenously administered PAC at the same dose [113]. Furthermore, another group modified the EVs derived from murine RAW 264.7 macrophages using PAC, showing a more than 50-fold increase in cytotoxicity in drug-resistant MDCKMDR1 (Pgp+) cells in vitro [114]. These last examples underline the predisposition of the colon to absorb EVs derived from different sources (Figure 3).

### 3.4. EVs in Pancreatitis Therapy

Pancreatitis, being an inflammatory condition, can give rise to diverse complications, included cardiac injury [115]. In the context of acute pancreatitis, this inflammatory state can lead to systemic complications such as hyperglycemia, hypoglycemia, and occasional ketoacidosis. MSCs can play a crucial role in mitigating these effects through their paracrine secretion of bioactive molecules [116,117]. These bioactive molecules are mostly delivered by MSC-EVs transporting growth factors, cytokines, chemokines, mRNA, miR, lncRNA, etc. [118]. Recent evidence has demonstrated the recovery of pancreas inflammation by inhibiting acinar cell apoptosis and controlling systemic inflammatory responses. This approach facilitates the repair of pancreatic tissue and contributes to the resolution of inflammation in the pancreas [119]. Li, S. et al. have demonstrated the enhancement of MSC-EVs in cell viability, mitigation of inflammation, and reduction in the expression of pyroptosis-related proteins in caerulein-stimulated pancreatic acinar cells, with a restoring effect on cell functions. The same study has proved the reduction of pancreatic acinar cell pyroptosis, in vivo, with a consequent decrease in the inflammatory response and oxidative stress in chronic pancreatitis, via the intraperitoneal and intravenous administration of MSC-EVs [120]. Furthermore, Wang et al. reported that the overexpression of the Klotho protein in MSC-derived exosomes has been effective in attenuating the severity of pancreatic inflammation in caerulein-stimulated AR42J cells [121]. The Klotho protein, naturally expressed in the pancreas, plays a crucial role in the digestive enzyme secretion from pancreatic acinar cells [122]. Other studies have corroborated these findings by demonstrating a decrease in the expression of IL-6 and TNF-α in acinar pancreatic cells treated with MSC-EVs overexpressing Klotho compared to control groups [123].

**Table 3:** Overview of different bio-technological EV-based preparations used in several functional GIDs. ↑ increase expression ↓ decrease expression.

Organ	Pathology	Biotechnology EV-Based Preparation	Targets and/or Pathways	Bibliography
Liver	Fatty liver and inflammation	EVs derived from intestinal microbiota: reduction in adipose dysfunction and inflammation	TLR2 ↑ TLR4 ↓	[89]
	MASLD/ MASH	EVs derived from Huc-MSCs (combined with Nilotinib): Reduction in liver fibrosis, liver steatosis and inflammation; liver reparation and regeneration	FTO ↓ TGF-β1 ↓ ERK1/2 and Bcl-2 ↑ IKKB/NFkB/casp-9/-3 ↓ NLRP3 ↓	[90,91,94]
	MASLD	Enrichment of oleic acid in exosomes: reduction in liver fibrosis in MASLD	elongase-6/RIP-1 ↑	[44]
Stomach	Esophagitis reflux	EVs released by BMSC: - reduction in functional dyspepsia, IBS, and esophagitis reflux and - modulation of the immune response; inflammation reduction	H3K27me3	[99]
	Gastritis	Exosomes derived from vaccine milk functionalized by PEG: stable new oral delivery system of si-RNA	ONCOGENES ↓ PD1/PD-L1 inhibitory axis ↓	[100,102]
	Esophagitis fistula	Allogenic EVs from adipose tissue-derived stromal cells, co-administered with a thermo-responsive gel: new strategy in the therapy of esophagitis fistula	Epigenetic regulation	[103]
Colon	Colitis	EVs released by ginger: improvement of colitis in a mice model	IL-22 ↑ IL-18 ↑ IL-10 ↑	[106,107,108]
		EVs derived from cow’s milk enriched with paclitaxel: improvement of pharmacokinetics and reduction in drug toxicity	MAPK ↑	[112]
Pancreas	Pancreatitis	EVs released by MSCs: - repair of pancreatic tissue - reduction in pancreatic inflammation severity	VEGF ↑ IL-6 ↓ TNF-α ↓ BAX ↓ BCL-2 ↑ NF-kB in Nucleoprotein ↓ NF-kB in plasmaprotein ↑ NLRP3 ↓	[116,117,120,122,123]

## 4. Structural Gastroenteric Disease and EV-Based Therapy

Structural GIDs manifest from abnormalities in the gastrointestinal tract, resulting in improper functioning of an organ or internal structure. These disorders may exhibit symptoms that overlap with functional GIDs but tend to be more severe, including significant and prolonged changes in bowel habits, obstructed bowel movements, and rectal bleeding. Common structural GIDs encompass conditions such as gastroenteric cancers, Crohn’s disease, and IBD. Unlike functional GIDs, the diagnosis and treatment of structural GIDs often necessitate more extensive procedures, frequently requiring surgical intervention for rectification and treatment. Neglecting serious structural GIDs may exacerbate symptoms and give rise to additional complications. This discussion focuses on several examples that showcase the use of EVs as drug delivery nanocarriers for treating structural GIDs. In gastroenteric cancers, properly modified EVs function as a Trojan horse, proficient in inhibiting tumor proliferation, metastasis, and, moreover, eliciting an anti-tumor immune response. The reported examples underscore the effective therapeutic potential of EVs, offering a valid alternative to conventional disease management approaches of structural GIDs.

### 4.1. Inflammatory Bowel Diseases

IBD, such as Crohn’s disease (CD) and ulcerative colitis (UC), are chronic conditions characterized by an inflammatory state of the gastrointestinal tract affecting more than 3.5 million people, whose incidence is increasing worldwide [124]. The current therapies do not guarantee the complete remission from the pathology with the consequent development of side effects due to the dose increase changes in therapy [125]. The EV treatments for colitis can be classified into two categories based on EVs’ provenience: microbiota, immune cells, stem cells, and ingesta. In this last decade, several anti-inflammatory miR-146 have been loaded into MSC-EVs for the treatment of colitis in mice models, considering their important role in the NF-kB activation of the TNF-receptor gene transcription associated with the factor 6 and IL-1 receptor in colon tissue [126]. Depletion of the microbiota is currently considered one of the possible causes of the evolution of IBD [77]. Both host derived EVs and BEVs, specifically OMVs, play a role in intestinal homeostasis. Several studies have demonstrated the role of miR-200-3p and miR-181b-5p delivered by EVs in regulating microbiota composition. EVs derived from normal feces have been shown to reverse intestinal dysbiosis in IBD subjects and restore the intestinal barrier, offering a new treatment for colitis. This effect is mediated by the enhancement of M2 polarization, inhibition of inflammation via PRKCD suppression, and activation of p-AKT [127]. BEVs also play a significant role in IBD, particularly in evaluating immune responses and modulating the intestinal barrier [128]. Additionally, BMVs isolated from normal intestinal microbiota have been shown to reverse the dysbiosis observed in colitis by inhibiting pathogenic populations. Although fecal microbiota transplantation is discussed as an advanced therapy to repair the microbiota in IBD patients, it can sometimes increase relapse rates. In contrast, membrane vesicle transplantation appears to be a more controllable, less risky, and readily available alternative for these patients [38].

### 4.2. Gastrointestinal Cancers

#### 4.2.1. EVs in HCC therapy

HCC is the most common type of primary liver cancer, and can occur in subjects with chronic liver disease, such as cirrhosis due to MASLD, hepatitis B (HBV), or hepatitis C (HCV) infection. Recently, several studies have shown that EVs secreted by HCC cells play a crucial role in HCC progression and metastasis [129]. Dwivedi et al. show the correlation of CD38 expression and response to anti-PD-1/PD-L1. CD38 is a multifunctional trans-membrane protein and is abnormally overexpressed in various tumors, which is associated with cancer progression [130]. The expression of the CD38 gene has been identified as a prognostic marker in HCC, particularly in relation to the pro-inflammatory state [131]. CD38 in the tumor microenvironment serves as an indicator of the response to anti-PD-1/PD-L1 immunotherapy in HCC [132]. Anti-PD-1/PD-L1 therapies have demonstrated efficacy in preventing immune evasion by tumor cells, making them a potent approach for various cancers, including HCC. However, despite the success of anti-PD-1/PD-L1 therapies, a significant number of patients become refractory to these treatments over time. This refractoriness limits the long-term effectiveness of anti-PD-1/PD-L1 therapies [133]. J. Deng et al. [134] have investigated the use of bone marrow MSC-EVs as a delivery system to convey CD38 siRNA (siCD38) (EVs/siCD38) to HCC cells. Their results have demonstrated that CD38 is upregulated in HCC cell-bearing mice with resistance to PD-1/PD-L1 inhibitor, and EVs/siCD38 reversed the resistance to PD-1/PD-L1 inhibitor in mice. This therapeutic approach holds the potential to not only limit the growth of HCC but also impede its metastasis. In a similar vein, C. He et al. [135] have developed EVs [136] as delivery vehicles for CRISPR-Cas9, aiming to reverse the therapy resistance of sorafenib by targeting key genes and cancer stem cells. Sorafenib is currently a highly recommended drug for HCC treatment; however, resistance to sorafenib is increasingly being reported [137,138]. Cancer stem cells (CSCs), representing a rare subpopulation within the tumor, are considered pivotal as they confer resistance to conventional cancer therapies [139,140] and are challenging to precisely eradicate due to their heterogeneity and plasticity. In this context, the surface of epithelial cell-derived EVs (HLC9-EVs) has been engineered using the human antibody HN3 to enhance the specific homing of these nanovectors to GPC3+, a protein overexpressed on the surface of liver cancer cells Huh-7. This approach has been reported in several chimeric antigen receptor T cell (CAR-T) studies [141,142]. The obtained HLC9-EVs have been loaded with sgIF to silence IQGAP1, a protein responsible for reactivating Akt/PI3K signaling in sorafenib resistance, and FOXM1, a self-renewal transcription factor in CSCs attributed to sorafenib resistance. This combination has been used in conjunction with sorafenib, demonstrating an effective synergistic anticancer effect in both in vitro and in vivo conditions.

#### 4.2.2. EVs in Pancreatic Cancer Therapy

The pancreatic gland serves a dual role in the body. Primarily, it is responsible for aiding in digestion by secreting digestive enzymes into the small intestine. Additionally, the pancreas plays a crucial role in regulating glucose levels in the bloodstream. It accomplishes this by producing the hormones, insulin and glucagon, which work together to maintain glucose homeostasis in the body. Insulin helps lower blood sugar levels by facilitating the uptake of glucose into cells, while glucagon acts to raise blood sugar levels by stimulating the release of stored glucose from the liver. Overall, the pancreas plays a vital role in both the digestive and endocrine systems, ensuring proper digestion and regulation of blood glucose levels. Pancreatic cancer (PC) ranks as the seventh most common cause of death among oncological diseases [143]. Treatment typically involves a combination of approaches, including surgery (such as Whipple procedure, total pancreatectomy, or distal pancreatectomy), chemotherapy (often combined with targeted therapy), and chemoradiation [144]. In recent years, the engineering of EVs has advanced, leading to the development of new nanosystems with applications in both diagnostics and therapeutics, such as the imaging-guided photothermal therapy (PTT) for cancers. A novel delivery system utilizing cronocaine dye (CR)-modified EVs for PTT has demonstrated significant photothermal activity. CR exhibits the potential utility for near infrared (NIR) imaging and theranostic applications. The combination of CR and EVs exhibits strong NIR absorption, excellent photothermal activity, good biological compatibility, and strong active tumor-targeting capability [145].

Similar to other types of cancers, miRNAs have been found to possess suppressive effects on the proliferation of, and invasion by, PC cells. One vehicle used as a drug delivery system in PC is EVs derived from human umbilical cord mesenchymal stromal cells (HUCMSCs). In particular, Ding et al. explored HUCMSC-derived EVs as a vehicles for miR-145-5p. They demonstrated that the delivery of miR-145-5p promoted apoptosis in vitro. Moreover, in an in vivo, xenograft tumor model, overexpression of miR-145-5p resulted in a reduction in tumor growth [146]. Furthermore, using exosomes derived from the same source but loaded with hsa-miR-128-3p was shown to suppress the proliferation, invasion, and migration of PANC-1 cells in vitro by targeting Galectin-3 [147]. Additionally, HUCMSC-derived exosomes were loaded with KRAS^G12D^ targeting siRNA via electroporation. This combination resulted in the reduced proliferation, migration, and viability of the KRAS^G12D^-PANC-1 cell line [148]. In the same study, Draguet et al. further investigated the endogenous loading of HUCMSC-EVs with doxorubicin (DOXO). They discovered that the rapid uptake of these loaded EVs in PANC-1 cells induced apoptotic cell death more efficiently than free DOXO. Their findings suggest that both methods could serve as effective therapeutic vehicles for delivering DOXO to PC cells [148]. S. Araujo-Abad et al. utilized small EVs derived from the RWP-1 cell line and loaded them with two chemotherapeutic drugs, temozolomide or EPZ015666, aiming to enhance the effectiveness of chemotherapy for treating PDAC, the most common aggressive form of PC. They observed a greater antiproliferative effect of temozolomide-loaded EVs compared to EPZ015666-loaded EVs when used in a PC cell line [149]. In a comparative investigation of various methods for loading EVs with chemotherapeutic agents, namely electroporation, sonication, and incubation with paclitaxel (PTX) and gemcitabine (GEM), it was demonstrated that sonication achieved superior efficiency in drug loading compared to incubation and electroporation. Additionally, EVs derived from the hTERT-HPNE cell line and incubated with PTX (HI-PTX) exhibited significantly increased cytotoxicity towards PDAC cells, employing clathrin-mediated endocytosis [150].

#### 4.2.3. EVs in GC Therapy

GC ranks as the third leading cause of cancer-related mortality globally. Like many other types of cancer, GC exhibits the upregulation of several genes, although only a handful are regarded as viable biomarkers. Among these, Cadherin 17 (CDH17) stands out as one of the most significantly upregulated genes in GC, with documented roles in promoting tumor progression, invasion, and metastasis in affected patients. Prior investigations have underscored CDH17 as a highly sensitive and specific marker for GC, with approximately 70% of GC cases expressing CDH17 at varying levels [151]. Indeed, CDH17 represents a promising target for GC therapy. P. Xia et al. [152] conducted a study where they isolated EVs from human embryonic kidney (HEK-293) cells and employed genetic engineering techniques to incorporate nanobodies into EVs, aiming to enhance their tumor-targeting capabilities against CDH17-positive cancers. Nanobodies are single-domain antibody fragments derived from heavy-chain-only antibodies found in camelids (e.g., camels and llamas). These nanobodies exhibit notable characteristics such as high target specificity, strong target affinity, effective tissue penetration, low inherent toxicity, and greater ease of genetic engineering compared to traditional full-length antibodies [153]. The engineered EVs were further loaded with Indocyanine green (ICG) dye and/or the anti-cancer drug, dinitroazetidine derivative RRx-001 (RRx-001), resulting in ICG/EVs and RRx-001/EVs, respectively, or a combination of both (ICG-RRx-001/EVs), serving as a blocker for the CD47/Signal Regulatory Protein Alpha (SIRPα) axis. While ICG is FDA approved, its clinical utility is constrained by poor water stability and off-target effects. To address these challenges, the authors encapsulated ICG into nanobody-engineered EVs for PTT applications [154]. RRx-001 was chosen in combination with PTT due to its outstanding preclinical performance. The study findings demonstrate that ICG/EVs enable rapid tumor imaging in a CDH17-positive GC model and induce significant antitumor PTT effects upon irradiation. RRx-001/EVs exhibit notable inhibition of GC tumor growth. Remarkably, a single administration of the complete nanoformulation almost entirely eradicated GC tumor growth in both cancer cell and patient-derived GC models.

Dysregulation of m6A modulators is recognized to contribute to tumor initiation, progression, metastasis, and resistance to anticancer therapies. YTH N6-methyladenosine RNA binding protein 1 (YTHDF1) is a critical m6A reader protein implicated in GC development. Various small-molecule inhibitors and RNA interference (RNAi) systems have been developed to target dysregulated m6A modulators, demonstrating significant efficacy in suppressing tumor progression and restoring antitumor immunity. Q. You et al. [155] engineered small EVs with high CD47 expression and cyclic arginine–glycine–aspartic acid (c(RGDyC)) modification to deliver short RNAi against YTHDF1 for GC treatment via epigenetic and immune regulation. The authors observed that this delivery nanosystem effectively reduced YTHDF1 expression, resulting in the inhibition of GC progression and metastasis. This effect was achieved by impeding the translation of FZD7 and deactivating the Wnt/β-catenin pathway in an m6A-dependent manner. Furthermore, the engineered EVs expressing CD47 competitively bind with signal regulatory protein α, thereby enhancing the phagocytosis of tumor cells by tumor-associated macrophages.

#### 4.2.4. EVs in CRC Therapy

CRC is a common type of cancer worldwide affecting the colon or rectum. The study conducted by S. Wu et al. introduces a promising therapeutic approach for CRC using folic acid-modified exosome–liposome hybrid nanoparticles loaded with ALKBH5 mRNA. The research demonstrates the effectiveness of these hybrid nanoparticles in significantly inhibiting the progression of CRC in preclinical tumor models. The underlying mechanism involves the modulation of the ALKBH5/JMJD8/PKM2 axis and the inhibition of glycolysis. Specifically, ALKBH5, an enzyme responsible for regulating m6A RNA methylation, is targeted in this novel therapeutic strategy. By applying folic acid-modified exosome–liposome hybrid nanoparticles loaded with ALKBH5 mRNA, the study presents a unique approach for CRC treatment. The modulation of the ALKBH5 pathway plays a crucial role in regulating RNA methylation patterns, ultimately influencing the progression of CRC [156].

The work by Phuong et al. delves into the utilization of exosomes derived from breast and CRC cells to enhance the uptake of nano-amorphous aspirin through both clathrin-dependent and independent endocytosis pathways. This investigation demonstrates the efficient delivery of aspirin to tumor sites in vivo, suggesting the potential of exosomes as carriers for therapeutic agents [157]. Similarly, Van et al. isolated EVs from CT26 CRC cells and 4T1 murine breast cancer cells and loaded them with doxorubicin (DOX) through electroporation. The results highlight the ability of these EVs to target CRC, with remarkable differences in uptake and penetration between CT26-derived EVs and 4T1 cell-derived EVs [158].

In the realm of CRC gene therapy, M. Hosseini et al. explored the use of CRC-derived EVs (CEXs) from CT26 loaded with miR-34a mimics. The CEX-miR-34a exhibits a reduction in invasion, angiogenesis, and immune evasion-related gene expressions in CRC. This intervention leads to prolonged survival in mice with colon cancer, demonstrating the potential of EVs as carriers for therapeutic miR cargo in the context of CRC gene therapy [159].

In a notable study focusing on novel oral delivery systems, EVs derived from natural sources have been employed. Grapefruit-derived EVs, loaded with methotrexate (MTX), were designed to target intestinal macrophages [160]. This approach resulted in a significant reduction in MTX toxicity and improved therapeutic effects in mouse colitis models. The use of natural sources, such as grapefruit-derived EVs, showcases the potential of EVs in the creation of innovative and targeted drug delivery systems. Similarly, milk-derived EVs (MEVs) have been explored for their advantageous properties. MEVs, characterized by a bilayer lipid membrane, facilitate easy crossing of the blood–brain barrier and cell membranes, ensuring the targeted delivery of cargo. Oral administration of MEVs was found to be stable in the gastrointestinal tract and demonstrated effectiveness in reducing the primary tumor burden in mouse models of CRC and breast cancer [160,161,162].

Another study reports on HEK293T cell transfection with a combined Her2-mCherry plasmid. These genetically engineered cells secrete exosomes expressing the Her2-LAMP2 fusion protein. The exosomes were then loaded with both 5-fluorouracil (5-FU) and a miR-21 inhibitor (miR-21i), creating a co-delivery system. This innovative system demonstrated efficacy in reversing drug resistance and significantly enhancing the cytotoxicity of 5-FU-resistant colon cancer cells. The use of exosomes for the co-delivery of therapeutic agents represents a promising strategy to overcome drug resistance and improve treatment outcomes in CRC [163]. Carboxylic acid-modified MUC1 aptamer (5TR1) has been covalently attached to the surface of MSC-derived exosomes, resulting in modified exosomes with higher tumor accumulation. These modified exosomes inhibited CRC growth in a mouse model [164]. Exosome-based hybrid nanostructures (EHNs) have been created by combining cancer cell-derived exosomes with magnetic nanoparticles and folic acid. These EHNs were loaded with the anticancer drug DOX. The application of alternating magnetic fields was found to enhance the CRC cell-killing effects and DOX cytotoxicity [165]. The afore mentioned studies concerning the treatment of structural GIDs wit EVs based nanoformulations have been summarized in Table 4.

## 5. Clinical Trials

To conclude, an overview of clinical trials registered at ClinicalTrials.gov pertaining to the investigation of EVs for diagnostic/prognostic and/or therapeutic purposes in gastroenteric tract diseases/conditions is reported in Table 5. A total of 18 registered clinical trials (2 withdrawn trials and not reported) were identified through a search using the keywords “extracellular vesicles” and “gastrointestinal diseases”. The majority of the identified trials were focused on exploring EVs for the identification of biomarkers, primarily through liquid biopsy methods. Among them, only four focused on the therapeutic use of EVs in gastroenteric tract diseases. These trials primarily utilized EVs derived from MSCs from various tissues, carrying their endogenous therapeutic cargo. Notably, none involved the use of engineered or modified EVs with exogenous components for the therapeutic purposes of GIDs. This suggests that the development of modified physiological EVs for clinical use is still in its early stages.

## 6. Conclusions and Perspective

The review showcases the considerable potential of EVs as biomarkers and nanocarriers for therapeutic agents in treating GIDs. These studies highlight the versatility of EVs in delivering treatments for various GIDs, promising better treatment outcomes. Despite the challenges, the review emphasizes the potential of EVs in revolutionizing gastroenterological therapeutics. Their role as delivery vehicles offers therapeutic hope in both functional and structural GIDs. From the medical point of view, it is evident that further clinical investigations are crucial to fully assess the safety and efficacy of these approaches in human patients. Challenges such as ethical considerations, large-scale production issues, isolation and purification complexities, drug loading efficiency, storage concerns, and the inherent heterogeneity of EVs pose hurdles in their clinical translation. Continued research and advancements in EV technology are deemed crucial for developing innovative therapeutic strategies in gastroenterology. Further clinical investigations will be essential to address the mentioned challenges for the successful translation of EV-based therapies into the clinical practice of GIDs.

## Figures and Tables

**Table 1:** Classification of EVs secreted by mammalian cells on the basis of their definition, size, genesis, and expression markers.

Name	Category	Size (nm)	Origin	Markers	Bibliography
Exosomes	Small EVs	30–120	Multivesicular endosomes and amphisomes	CD63 Syntenin 1 CD81	[9,10]
Small ectosomes	Small EVs	50–200	Ectosome	TSG101 CD9 ARDC1 CD 147	[11,12,13]
Microvesicles	Small to large	200–1000	Ectosome	Annexin A1 Annexin A2 α-Actin 4	[14]
Apoptotic Body	Small to large	100–5000	Apoptosis	Annexin V	[15]

**Table 2:** Different EV biomarkers reported for organs of the gastroenteric apparatus and related pathologies.

Organ	Pathology	EVs Markers	Targets and/or Pathway	Bibliography
Pancreas	Pancreatitis	miR-155	SOCS1 regulation	[55,83]
	PDAC	miR-192-5p	Epigenetic regulation	[57]
		miR-21	MMP2, MMP9, and VEGF regulation	
		miR-25	Cell proliferation promotion	
		miR-16	Post-transcriptional expression of Bcl-2 regulation	
	PC	MUC2, MUC4, MUC5AC, MUC6, MUC13	Elevated pathway regulation determining the severity of PC	[58,59,60,61,62]
		EGFR, GPC1, WNT-2, EpCAM, MUC1	Aberrant protein	
		Das-1	3′-sulfated Lewis A/C recognition	
		STMN1	Cell proliferation inhibition	
		TSP1	Participation in the differentiation of Th17 cells	
		TSP2	Osteoblasts regulation	
		ZEB1	EMT induction	
		EphA2	Cell migration	
		S100A4	Src and FAK activation	
		PSCA	Thymic lymphocyte differentiation, maturation, and activation	
		HOOK1	Re-arrangement of the cytoskeleton	
		PTPN6	SP1/MAPK signaling pathway inhibition	
		FBN1	Immune cell infiltration in tumors increases	
Stomach and Esofagous	GERD and Gastritis	miR-29a-3p	EMT, ZEB 1 and ZEB2 regulation	[63]
	Atrofic Gastritis	miR-122-5p	Targeting CTDNEP1/LPIN1	[67]
	GC	BARHL2	Cell differentiation	[69]
		LncUEGC1	Unknown	[70]
		LncRNA HOTTIP	Proliferation promotion and apoptosis inhibition	[71]
		FZD10	Increasing of Ki-67 expression via Phospho-ERK1/2	[20]
Colon	IBD	Annexin A1	Inflammation reduction	[75,84]
		miR-200b-3p NEAT lncRNA	Protein expression regulation	[76,77]
		PSMA7	Role in proteosomal activity	[73]
		lncRNA H19	Unknown	[72]
	CRC	FZD10	Increasing of Ki-67 expression via Phos-pho-ERK1/2	[20]
		CD147	Glycolipid metabolism reprogrammation	[18]
		A33	Cell surface targeting for antibody-based therapy	[81]
		MARCKSL1	Role in the Immune System	[82]
		Wnt	Role in via β-catenin/SOX2	[79]
		CPNE3	Promotion of cell proliferation via the PI3K/AKT pathway	[80]

**Table 4:** Overview of the modifications of EVs and their possible application in structural GIDs.

Organ	Pathology	EV-Based Preparations and Applications	References
liver	HCC	EVs released by BM-MSCs as a delivery system to convey siCD38 to HCC cells: potential limitation of HCC growth and spread	[134]
		Engineered EVs mediated CRISPR-Cas9 to reverse the therapy resistance of sorafenib: the suppression of HCC cancer stem cells HLC9-EVs engineered with HN3: increasing in specific homing	[135,141,142]
pancreas	PDAC PC	Delivery system based on CR-modified EVs for PTT	[145]
		EVs derived from HUCMSCs loaded with miR-145-5p: apoptosis and cancer growth inhibition	[146]
		EVs derived from HUCMSCs loaded with hsa-miRNA-128–3p: apoptosis and cancer growth inhibition	[147]
		EVs derived from HUCMSCs loaded with KRAS G12D targeting siRNA: a reduction in the proliferation, migration, and viability in PANC-1 cells	[148]
		HUCMSC-EVs with DOXO: the rapid uptake in PANC-1 cells induced apoptotic cell death more efficiently than free DOXO	
		RWP-1-derived EVs loaded with temozolomide and EPZ015666: a greater antiproliferative action of EVs encapsulating on the PC cell line EVs derived from the hTERT-HPNE cell line and incubated with PTX: PDAC cell death via clathrin-mediated endocytosis	[149,150]
stomach	GC	EVs from HEK-293 cells targeting CDH17-positive GC cancers by the engineered nanobodies into EVs with genetic engineering techniques	[151,152,153,154]
		Engineered small EVs with high CD47 expression and c(RGDyC) modification to deliver short RNAi against YTHDF1: a novel therapeutic strategy for GC via epigenetic and immune regulation	[155,166]
colon	IBD	EVs released by MSCs and loaded with miR-146: a new strategy in the treatment of IBD in mouse models	[126]
		EVs and bacteria-derived membrane vesicles (OMVs) derived from normal feces: restore the intestinal barrier in IBD subjects	[38,127,128,167]
		Exo containing PSMA7 derived from saliva: an important protein biomarker for IBD	[168]
	CRC	Folic acid-modified exo–liposome hybrid nanoparticles loaded with ALKBH5 mRNA: a novel therapeutic strategy for CRC	[156]
		EVs derived from CT26 and loaded with miR-34a mimics: a new strategy for CRC gene therapy in mouse model	[159]
		EVs derived from milk: a reduction in primary tumor burden in mouse models of CRC and breast cancer	[161,162]
		Exosomes secreted from HEK293T transfected with Her2-mCherry plasmid and loaded with both 5-FU and miR-21i: reversing the drug resistance in CRC cells	[164,165]
		Exosomes secreted from MSCs modified with 5TR1 or with magnetic nanoparticles, folic acid, and DOXO: the inhibition of CRC growth in mouse models	[151,152,153,154]

**Table 5:** Registered clinical trials (clinicaltrials.gov, accessed date 4 March 2024) related to diagnosis, follow-up, and therapy by using extracellular vesicles as biomarkers or natural carriers of therapeutic cargo.

Study Title	Condition or Disease	EVs for Biomarker Detection	EVs for Therapy	ClinicalTrials.gov Identifier
Portal Hypertension in Non-alcoholic Fatty Liver Disease: Association with Cardiovascular Risk and Identification of non-invasive biomarkers	NAFLD	Identification of non-invasive biomarkers, including EVs, and of the presence and severity of portal hypertension by liquid biopsy		NCT04191044
Liver Health and Metabolic Func-tion in People with Obesity	NAFLD	Amount, content, and function of EVs from adipose, liver, and blood tissue collected before and after ~20% weight loss and at the time of surgery		NCT03701828
Role of Immune System in Obesity-related Inflammation and Cardiometabolic Risk	NAFLD MASLD	Investigation of the signaling between cells and organs examined by isolating the exosomes from the blood, subcutaneous and omental adipose tissue of patients scheduled for gallbladder, inguinal hernia, hysterectomy, or myomectomy surgery		NCT01104220
Muscles in Liver Diseases	Liver Diseases	Identification of circulating mediators, including EVs, that could be responsible for the complications of liver disease: EVs released by the muscle and acting on different organs		NCT04758793
Safety of Injection of Placental MSC-Derived Exosomes for Treatment of Resistant Perianal Fistula in Crohn’s Patients	Perianal Fistula Crohn’s Disease	Identification of inflammatory markers in exosomes through laboratory workup, including CRP, IL-6, TNF-a, calprotectin	Evaluation of the safety and clinical efficacy of injected exosomes	NCT05499156
Study of ExoFlo for the Treatment of Perianal Fistulas	Perianal Fistula Crohn’s Disease		Ex Vivo Culture-expanded Adult Allogeneic Bone Marrow MSC-derived EVs for the Treatment of Perianal Fistulizing Crohn’s Disease Local injection of normal saline	NCT05836883
Study of ExoFlo for the Treatment of Medically refractory Crohn’s Disease	Crohn’s Disease		Intravenous ex vivo culture-expanded adult allogeneic bone marrow MSC-EVs in subjects with medically refractory Crohn’s disease who do not respond to monoclonal antibody-based therapy: evaluation of feasibility and efficacy	NCT05130983
Study to Evaluate the Epidemiology and the Characteristics “Omics” in Patients Recently Diagnosed of Inflammatory Bowel Disease in Spain (IBDomics)	Inflammatory Bowel Diseases	Characterization of the composition of serum EVs in newly diagnosed IBD patients, aiming to identify the molecular and cellular pathways involved in IBD development and pathogenesis correlation of the serum proteomic profile and the density and composition of serum EVs with an IBD phenotype		NCT03689257
Study of ExoFlo for the Treatment of Medically Refractory Ulcerative Colitis	Ulcerative Colitis Inflammatory Bowel Diseases		Intravenous ex vivo culture-expanded adult allogeneic bone marrow MSC-EVs in subjects with medically refractory Crohn’s disease who do not respond to monoclonal antibody-based therapy: evaluation of feasibility, safety, and efficacy	NCT05176366
Pancreatic Cancer Initial Detection Via Liquid Biopsy (PANCAID)	Pancreatic Cancer Chronic	Detection of pancreatic cancer in bio-banked samples, including EVs, of patients with histologically confirmed pancreatic lesions via liquid biopsy		NCT06283576
Impact of Graft Reconditioning with Hypothermic Machine Perfusion on HCC Recurrence After Liver Transplantation	Hepatocellular Carcinoma	Identification of microRNAs from liquid-biopsy-derived EVs, as tools of prognostic information on IRI favoring hepatocellular carcinoma recurrence		NCT06236568
Metronomic Capecitabine, Oxaliplatin and UGT1A1 Genotype-directed Irinotecan in Metastatic Pancreatic Cancer Patients	Metastatic Pancreatic Cancer	Identification of exosomal proteins secreted by EVs from plasma using mass spectrometry, at different times: at pre-dose, the end of oxaliplatin infusion, and the end of irinotecan infusion		NCT05929885
A Prospective Feasibility Study Evaluating EVs Obtained by Liquid Biopsy for Neoadjuvant Treatment Response Assessment in Rectal Cancer	Rectal Cancer	Tumor-EV detection and quantification for the response assessment and follow-up of patients with adenocarcinoma of the rectum		NCT04852653
Contents of Circulating Extracellular Vesicles: Biomarkers in Colorectal Cancer Patients	Colorectal Cancer	Exploitation via liquid biopsy of circulating tumor-exosomes containing markers, namely specific miRNAs, useful as biomarkers of the early prognosis of patients with colon cancer		NCT04523389
Exosome-based Liquid Biopsies for Upper Gastrointestinal Cancers Diagnosis	Gastric Cancer Esophagus Cancer	Identification and quantification via proteomic analysis of specific proteins as biomarkers for upper gastrointestinal tumors Multicentre and retrospective study		NCT06278064
Prospectively Predict the Efficacy of Treatment of Gastrointestinal Tumors Based on Peripheral Multi-omics Liquid Biopsy	Advanced Gastric Adenocarcinoma	Validation of 4 plasma EV-derived proteins and their combination as a signature score for robustly predicting immunotherapeutic outcomes Prospective study		NCT04993378
